# Hepatectomy inside the Portal Ring in a Patient with Absence of Portal Vein Bifurcation: A Case Report

**DOI:** 10.70352/scrj.cr.25-0637

**Published:** 2026-01-23

**Authors:** Atsuhito Takagi, Daigoro Takahashi, Atsuyuki Maeda, Yuichi Takayama, Takamasa Takahashi, Hiroki Aoyama, Takahiro Hosoi

**Affiliations:** Department of Surgery, Ogaki Municipal Hospital, Ogaki, Gifu, Japan

**Keywords:** abdominal pain, absence of portal bifurcation, gallbladder carcinoma, liver resection, portal ring, portal vein, tomography

## Abstract

**INTRODUCTION:**

Absence of portal vein bifurcation (APB) is a rare congenital anomaly in which the main portal vein does not bifurcate at the hepatic hilum. After giving off posterior and anterior sectoral branches, the trunk curves ventrally around the middle hepatic vein—forming a so-called “portal ring”—and finally enters the umbilical portion of the left portal vein. Hepatectomy performed inside this ring risks compromising perfusion if the portal trunk is injured. We report a case in which anatomical segmentectomy (S4a+S5) was performed for the gallbladder carcinoma with this portal vein anomaly.

**CASE PRESENTATION:**

A 69-year-old man presented with 1 month of epigastric pain. Imaging revealed gallbladder carcinoma invading the gallbladder bed and liver parenchyma, together with APB confirmed by 3D reconstruction. Based on preoperative simulation, we performed anatomical resection of segments 4a and 5 with lymphadenectomy. Intraoperative ultrasonography verified the portal ring anatomy; the anterior Glissonean pedicle (G5) was ligated and the demarcation line was followed for parenchymal transection under intermittent Pringle maneuver (total ischemic time 70 minutes). Operative time was 6 hours 3 minutes, and blood loss 170 mL. Pathology showed nodular gallbladder carcinoma (UICC 8th: pT3a, pN1 [#8p, #12a], cM0; Stage IIIB) with negative margins. The postoperative course was uneventful and the patient was discharged on day 12.

**CONCLUSIONS:**

Preoperative 3D simulation and meticulous intraoperative ultrasonography enabled safe hepatectomy inside the portal ring without compromising portal perfusion. In APB, right-sided major hepatectomy may jeopardize global portal inflow; tailored anatomical resection (S4a+S5) achieved an R0 resection while preserving perfusion.

## Abbreviations

APB
: absence of portal vein bifurcation
CECT
: contrast-enhanced CT
MHV
: middle hepatic vein
PV
: portal vein

## INTRODUCTION

Absence of portal vein bifurcation (APB) is an extremely rare anomalous anatomy of portal vein (PV) branching. APB was first reported by Couinaud^1)^ and occurs at a certain rate according to Aggosou and Voyeme.^2)^ Some reports have shown that the prevalence of this anomaly is 0.03%–1.0%.^3–8)^ Embryologically, failure of vitelline venous development near the hepatic hilum results in a main portal trunk that does not bifurcate into right and left branches; instead, after giving off posterior and anterior sectional branches, it courses ventrally around the middle hepatic vein (MHV) as a “portal ring” before reaching the umbilical portion of the left PV.^1,9)^ Because the hepatic artery and bile duct develop subsequent to the portal venous system, APB may coexist with arterial and biliary anomalies, necessitating precise preoperative simulation.

For advanced gallbladder carcinoma (≥T3), anatomical liver resection is often indicated to achieve negative margins. However, in APB, major right hepatectomy approaches and manipulates the portal trunk within the portal ring and can compromise the global portal inflow. We therefore planned S4a+S5 anatomical resection inside the portal ring to balance oncological radicality and vascular safety.

## CASE PRESENTATION

**Fig. 1:**
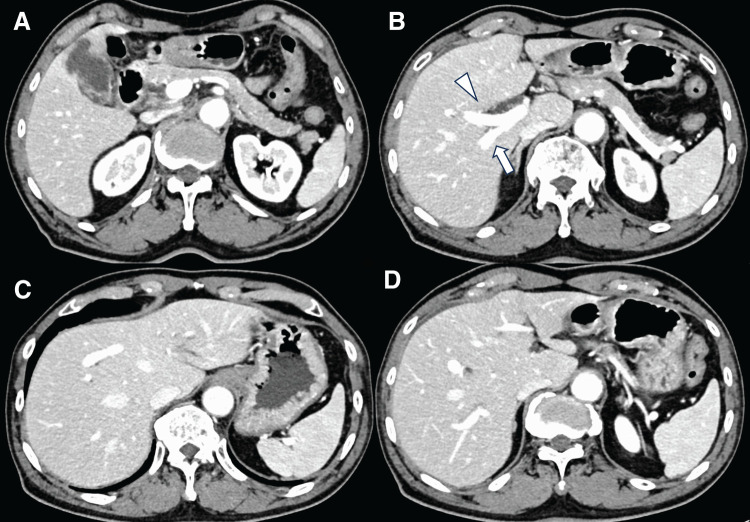
CECT revealed gallbladder carcinoma and the absence of a PV. (**A**) The CECT scan indicates the gallbladder that infiltrated the gallbladder bed and liver parenchyma. (**B**–**D**) The CECT scan shows the intrahepatic path of the main trunk of the PV. The portal ring runs through the right liver and inflows the left liver. The white arrowhead indicates the anterior PV and the white arrow indicates the posterior PV. CECT, contrast-enhanced CT; PV, portal vein

**Fig. 2:**
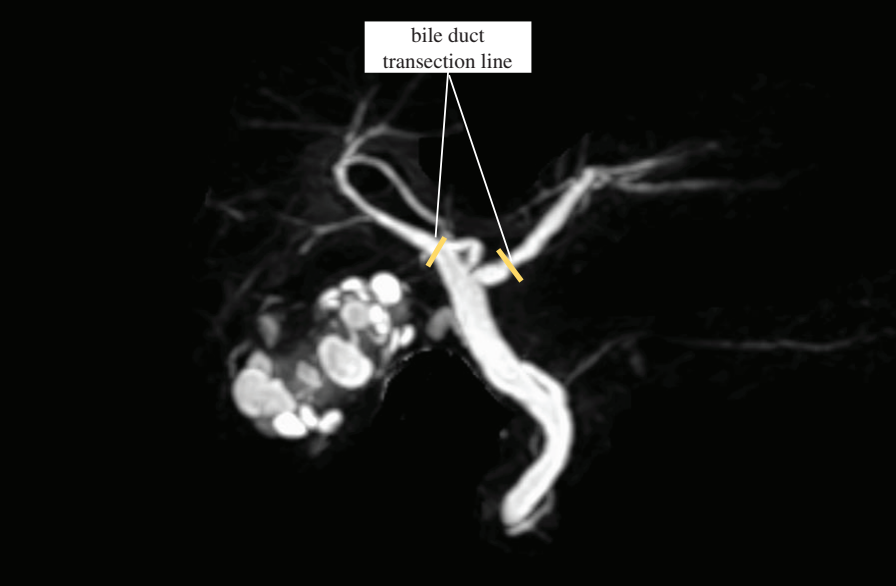
MRI showed that the right hepatic duct ran along the right branch of the portal vein and joined the left hepatic duct.

A 69-year-old man presented with epigastric pain persisting for 1 month. Ultrasonography, contrast-enhanced CT (CECT), and MRI revealed a 20-mm gallbladder tumor infiltrating the gallbladder bed and adjacent parenchyma (**Figs. 1A** and **2**).

**Fig. 3:**
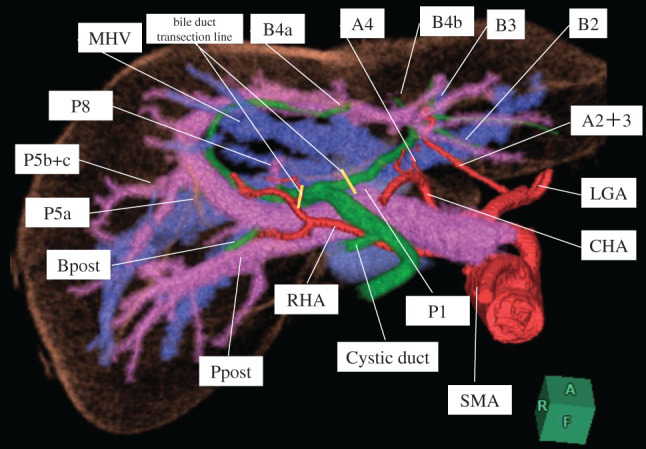
3D simulated image of the liver (orange), hepatic artery (red), portal vein (pink), bile duct (green), and hepatic vein (blue). The relevant intrahepatic vascular anatomy associated with the resection is demonstrated in the figure. CHA, common hepatic artery; LGA, left gastric artery; MHV, middle hepatic vein; RHA, right hepatic artery, SMA; superior mesenteric artery

**Fig. 4:**
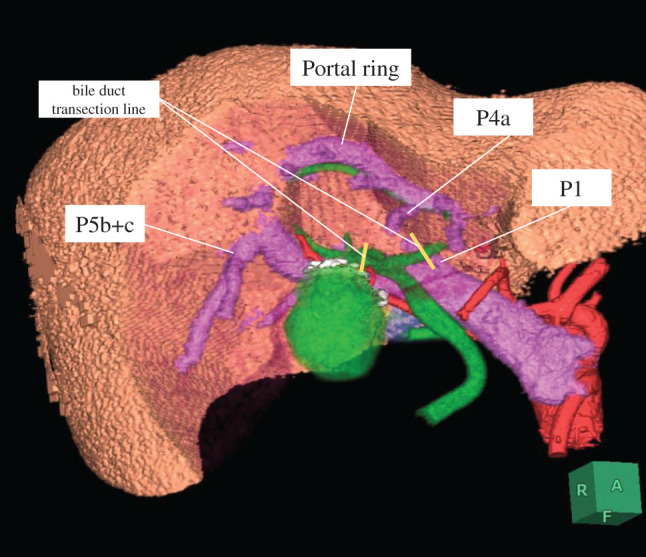
The liver dissection line is simulated by the 3D image of the liver (SYNAPSE VINCENT version 5, FUJIFILM, Tokyo, Japan). The portal vein branches exposed after liver resection are illustrated in the figure.

Additionally, CECT revealed the presence of a gallbladder tumor and an anatomical anomaly of the PV (**Figs. 1B**–**1D** and **3**). 3D reconstruction revealed the anatomical characteristics of the liver, PV, hepatic artery, and bile duct (**Fig. 3**). These preoperative examinations revealed that the main PV ran arcuately and gave rise to the posterior, anterior, medial, and lateral branches in each segment. The hepatic artery originated from the left gastric artery in segments 2 and 3, the common hepatic artery in segment 4, and the superior mesenteric artery in the right branch. Regarding the intrahepatic bile duct (**Fig. 3**), B1, B4a, and B5-8 joined the left hepatic duct following the course of the portal ring. However, B2, B3, and B4b joined the left hepatic duct independent of the PV course. The MHV passed through the portal ring and drained into the inferior vena cava. Based on these anatomical findings, S4a and S5 anatomical liver resection with hepatoduodenal ligament lymphadenectomy was planned (**Fig. 4**).

**Fig. 5:**
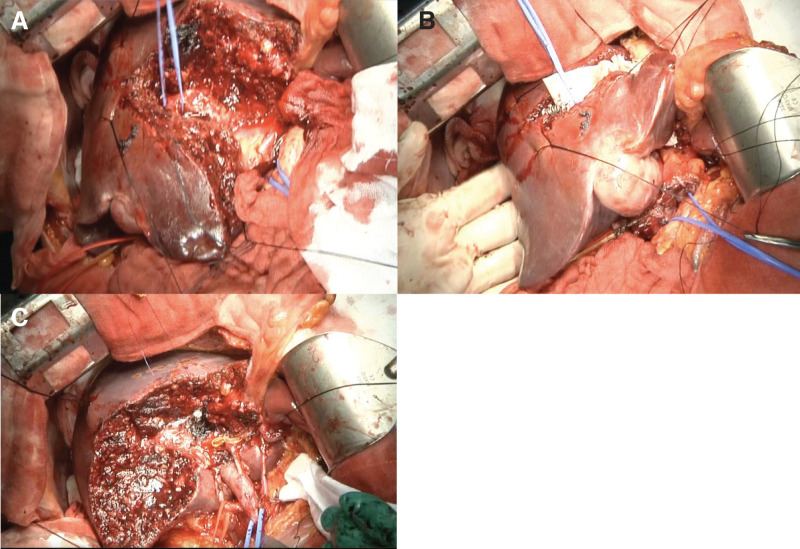
S4a+S5 liver resection in the patient with APB. (**A**) Liver dissection was started from the right side of the hepatic circumflex, and the G5b+c was exposed and taped. (**B**) G5b+c was clamped, and a demarcation line was observed on the right side of the liver. (**C**) The dissected cross-sectional view of the liver after removal showed a defect in the left branch of the portal vein. APB, absence of portal vein bifurcation

**Fig. 6:**
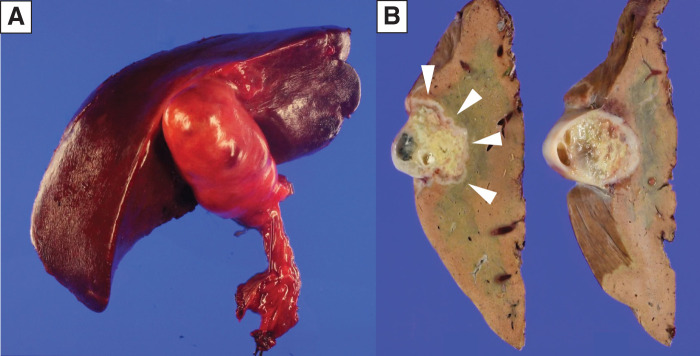
Specimen (**A**) The unfixed specimen showed an enlarged gallbladder with induration before formalin fixation. (**B**) The specimen showed a lesion that exceeded the wall of the gallbladder and infiltrated into the liver parenchyma after formalin fixation.

Intraoperatively, ultrasonography confirmed the portal ring and the umbilical portion anatomy. Although this finding was not discernible from the liver surface, intraoperative dissection at the site corresponding to the normal left PV revealed a fibrous remnant (cord-like structure). The Glissonean sheath was present as usual, and the vascular structures—hepatic artery and bile duct—were managed individually. Subsequently, a lymphadenectomy of the hepatoduodenal ligament was performed. Liver parenchymal transection was initiated from the right side of the round ligament under intermittent Pringle maneuver. After ligation of the Glissonean sheath of segment 4a including some small branches of the MHV, the anterior Glissonean pedicle was exposed, and the root of G5 was ligated (**Fig. 5A**–**5B**), producing a clear demarcation line for anatomical transection. Optical liver transection was performed along the demarcation line (**Fig. 5C**). Subsequently, intrahepatic cholangiojejunostomy was performed as indicated. Total hepatic inflow occlusion time was 70 minutes; operative time 6 hours 3 minutes; and blood loss 170 mL. Histopathological examination revealed nodular gallbladder carcinoma arising from the fundus of the gallbladder with negative margins and metastases in #8p and #12a nodes (UICC 8th edition: pT3a, pN1, cM0, pStage IIIB) (**Fig. 6A**–**6B**). The postoperative course was uneventful and the patient was discharged on POD 12.

## DISCUSSION

APB is an anomaly of the PV bifurcation, first described by Couinaud who described this anomaly as “a most unusual and dangerous anomaly.” Agossou-Voyeme reported that APB was a vascular malformation occurring at a certain frequency. Some reports have shown that the prevalence of APB is 0.03%–1.0% in cases of imaging studies and 1.0%–3.0% in autopsy cases. In terms of embryology, the portal venous system develops in the fifth week of embryogenesis through fusion and regression of the left and right vitelline and umbilical veins. Developmental failure of the vitelline veins near the hepatic hilum results in the absence of portal trunk bifurcation. This helps the intrahepatic portal branches to form an arch-like structure. These branches develop from the right PV and extend toward the left hepatic lobe. Consequently, the main portal trunk proceeds directly toward the right hepatic lobe without bifurcating into the left and right branches of the hepatic hilum. After branching into the posterior and anterior sectional branches, the main portal trunk turns leftward and runs transversely toward the umbilical portion of the PV ventral to the MHV. Couinaud referred to this trajectory as the “portal ring,” which circles the MHV. From an embryological perspective, the hepatic artery and bile duct develop after the portal venous system formation. PV anomalies often lead to anomalies in the hepatic artery and bile duct. Therefore, preoperative simulation is essential for successful procedures.

To date, 33 studies have described APB^1–35)^ Several reports have described 12 cases of systematic liver resection of malignant tumors. In the present case, 13 cases are summarized in **Table 1**.^13,14,19,20,24,27,28,30,31,33–35)^ Of the 13 cases, partial liver resections were performed in 2, Caudal lobectomy in 1, resection of subsegment in 2, sectionectomy in 2, and hepatectomy in 6. Among the hepatectomy cases, left and right hepatectomies were performed in 2 and 4 cases, respectively. Intraoperative diagnosis of APB is challenging due to the lack of macroscopic abnormalities in the liver. In 1994, Koh et al.^13)^ reported a case wherein the absence of APB was not recognized intraoperatively and the right PV was ligated and transected, causing cessation of portal blood flow to the entire liver and subsequent death. Cheynel et al.^20)^ and Teraoku et al.^30)^ reported cases wherein right hepatectomy was safely performed using detailed preoperative CT. Terasaki et al.^8)^ analyzed the anatomical features of the PV, bile duct, hepatic veins, and hepatic arteries in five cases of APB encountered at a single institution. That report provided detailed description of the course and branching patterns of the PV. To date, all reported cases have used surgical techniques to preserve the portal ring. In cases of right hepatectomy with APB, the branches of the PV located ventral to the main trunk at the liver surface were transected along with the vessels outside the portal ring as part of the systematic right hepatectomy. However, Teraoku et al.^30)^ have reported thrombosis of the PV exposed during the course of hepatic resection. Therefore, the reports indicate the risk of manipulation when approaching the main PV trunk in APB cases. Couinaud referred to the MHV instead of the right hepatic vein, which developed in the APB because the right hepatic vein drains only the hepatic parenchyma of the posterior section. In previously reported APB cases requiring hepatectomy, the MHV was located ventral to the portal ring in 7 cases and dorsal to it in 3 cases. Since S4a+S5 liver resection is the procedure that needs hepatectomy inside the portal ring and approaches the main trunk of the MHV and PV, the manipulation should be paid particular attention not to injure such vascular system. To our knowledge, no previous reports have described hepatectomy requiring parenchymal transection inside the portal ring. In cases such as ours, where the MHV traverses the portal ring, careful parenchymal transection must be performed while individually controlling the vascular branches arising from the main portal trunk within the ring, taking care to preserve the main drainage vein.

**Table 1:** Previous case reports of hepatectomy for absence of portal vein bifurcation

No.	Author	Year	Age	Sex	Diagnosis	Characteristics of the relationships with MHV	Operation
1	Koh^13)^	1994	45	Female	Caroli’s disease	N.D	Right hepatectomy
2	Hardy and Jones^35)^	1997	47	Female	Focal nodular hyperplasia (S4)	Dorsal	Segment 4 partial liver resection
3	Charney^14)^	1997	33	Female	Remnant of a choledochus	N.D	Left hepatectomy
4	Yamamoto^19)^	2000	61	Female	Hilar cholangiocarcinoma	Ventral	Left hepatectomy
5	Cheynel^20)^	2001	64	Male	Liver metastasis from colorectal carcinoma	Ventral	Right hepatectomy
6	Kotake^24)^	2003	59	Female	Cholangiocellular carcinoma	Ventral	Right posterior sectionectomy
7	Spampinato^27)^	2012	N.D	N.D	Liver metastasis from colorectal carcinoma (S8)	Ventral	Right hepatectomy
8	Ishikawa^28)^	2012	62	Male	Hepatocellular carcinoma (S5)	Ventral	Segment 5 resection
9	Teraoku^30)^	2016	61	Male	Hepatocellular carcinoma	Dorsal	Right hepatectomy
10	Tanaka^31)^	2017	60	Male	Hepatocellular carcinoma (S6)	Ventral	Segment 6 partial resection
11	Takei^33)^	2019	84	Male	Hepatocellular carcinoma (S1)	Ventral	Caudal lobectomy
12	Machado^34)^	2022	61	Male	Hepatocellular carcinoma (S6,7)	N.D	Right posterior sectionectomy
13	Takagi	2023	69	Male	Gallbladder carcinoma	Dorsal	Anatomical segmentectomy (S4a+5)

Gallbladder cancer is known to invade the adjacent organs, such as the liver and the hepatoduodenal ligament, because of its anatomical location and is frequently associated with lymph node metastases. Radical resection is the only treatment that enables long-term survival in patients with advanced gallbladder cancer. Patients classified as having T3 or deeper disease often require systematic liver resection, including liver S4a+S5 resection, right hepatectomy, or right three-sectional resection combined with extrahepatic bile duct resection. When aiming for R0 resection, carefully balancing between safety and prognosis and determining an appropriate surgical approach based on the tumor depth of invasion are essential. In this case, the patient had gallbladder cancer and liver invasion. However, due to the coexistence of the APB, right hepatic hepatectomy or right three-sectional resection would have resulted in transection of the main portal trunk, leading to cessation of blood flow to the entire liver, thereby compromising surgical safety. Liver S4a+S5 resection allowed the removal of the tumor infiltrating the gallbladder bed with an adequate surgical margin.

In our case, S4a+S5 anatomical resection achieved oncologically adequate margins for T3 gallbladder carcinoma while avoiding injury to the portal trunk inside the ring. Key safeguards included: (i) comprehensive preoperative 3D mapping of the portal, arterial, and biliary anatomy; (ii) intraoperative ultrasonography to verify the course of the portal ring and MHV; (iii) Glissonean-first pedicle control to define anatomical boundaries; and (iv) selective biliary reconstruction as required.

## CONCLUSIONS

Anatomical resection of segments 4a and 5 inside the portal ring provided an R0 resection for gallbladder carcinoma while preserving global portal perfusion. Meticulous preoperative 3D simulation combined with intraoperative ultrasonographic navigation is indispensable for performing safe hepatectomy inside the portal ring in patients with an absence of PV bifurcation.

## DECLARATIONS

### Availability of data and materials

The data sets used in the current study are available from the corresponding author upon reasonable request.
